# Isothermal Amplification Using a Chemical Heating Device for Point-of-Care Detection of HIV-1

**DOI:** 10.1371/journal.pone.0031432

**Published:** 2012-02-23

**Authors:** Kelly A. Curtis, Donna L. Rudolph, Irene Nejad, Jered Singleton, Andy Beddoe, Bernhard Weigl, Paul LaBarre, S. Michele Owen

**Affiliations:** 1 Laboratory Branch, Division of HIV/AIDS Prevention, National Center for HIV/AIDS, Hepatitis, STD, and TB Prevention, Centers for Disease Control and Prevention, Atlanta, Georgia, United States of America; 2 PATH, Seattle, Washington, United States of America; Rush University, United States of America

## Abstract

**Background:**

To date, the use of traditional nucleic acid amplification tests (NAAT) for detection of HIV-1 DNA or RNA has been restricted to laboratory settings due to time, equipment, and technical expertise requirements. The availability of a rapid NAAT with applicability for resource-limited or point-of-care (POC) settings would fill a great need in HIV diagnostics, allowing for timely diagnosis or confirmation of infection status, as well as facilitating the diagnosis of acute infection, screening and evaluation of infants born to HIV-infected mothers. Isothermal amplification methods, such as reverse-transcription, loop-mediated isothermal amplification (RT-LAMP), exhibit characteristics that are ideal for POC settings, since they are typically quicker, easier to perform, and allow for integration into low-tech, portable heating devices.

**Methodology/Significant Findings:**

In this study, we evaluated the HIV-1 RT-LAMP assay using portable, non-instrumented nucleic acid amplification (NINA) heating devices that generate heat from the exothermic reaction of calcium oxide and water. The NINA heating devices exhibited stable temperatures throughout the amplification reaction and consistent amplification results between three separate devices and a thermalcycler. The performance of the NINA heaters was validated using whole blood specimens from HIV-1 infected patients.

**Conclusion:**

The RT-LAMP isothermal amplification method used in conjunction with a chemical heating device provides a portable, rapid and robust NAAT platform that has the potential to facilitate HIV-1 testing in resource-limited settings and POC.

## Introduction

HIV-1 diagnostic tests are held to a high standard of performance, as diagnosis has a direct impact on patient care and reduction of transmission. Despite technological advances in the field of HIV diagnostics and the high sensitivity and specificity associated with most HIV diagnostic tests that are currently available, it is estimated that approximately 20% of HIV-infected individuals living in the United States remain undiagnosed [1]. Furthermore, testing sites have reported as many as 35 to 50% of individuals with an initial positive test result will not return for a confirmatory diagnosis if follow-up laboratory testing is required [2]. Rapid HIV antibody-based tests, which can be performed with minimal training and typically provide results in under 30 minutes [3], have facilitated HIV testing at the point-of-care and subsequently increased the numbers of individuals aware of their serostatus [4]. Rapid tests are currently a key component of HIV screening at the point-of-care (POC), significantly expanding the diagnostic capabilities of testing sites in developed countries, as well as resource-limited settings.

Despite the advances made by the widespread availability of rapid tests, all antibody-based tests for the detection of HIV exhibit some limitations. HIV-specific antibody typically begins to appear around three weeks post-infection, allowing for detection by most antibody-based assays within 3–6 weeks [3], [5]. The window of time prior to or during early seroconversion may lead to false-negative test results in recently infected individuals. Additionally, accurate diagnosis of infants born to HIV-infected mothers can be challenging if based solely on antibody positivity, since vertically transferred maternal antibodies may persist for 12–18 months after birth [6], [7]. For confirmatory diagnosis of early HIV infection or infant diagnosis, nucleic acid amplification tests (NAAT) are preferred, as HIV-1 RNA can be detected as early as 10–12 days post infection and HIV-1 DNA and/or RNA are definitive indicators of active infection [5]. In their current form, however, NAAT's are not feasible for POC testing, because they are time-consuming, expensive, and technically complicated. To date, the Aptima HIV-1 RNA assay (Gen-Probe, Inc., http://www.fda.gov/BiologicsBloodVaccines/BloodBloodProducts/ApprovedProducts/LicensedProductsBLAs/BloodDonorScreening/InfectiousDisease/UCM080466) is the only FDA-approved NAAT for the diagnosis or confirmation of HIV-1 infection and it is only suitable for laboratory testing.

To meet the needs of HIV-1 diagnosis at the POC, a rapid NAAT that can be performed with minimal training, limited equipment, and a relatively short turnaround time (<1 hour)is desirable [8]. The development of a rapid NAAT has proven to be especially challenging since the technology involved in simplifying the test procedure often equates to increased equipment and material costs [8]. Additionally, the reduction in technical complexity should not compromise test sensitivity and specificity. For increased applicability at the POC, an increasing number of novel isothermal amplification techniques have been developed [9]. Isothermal amplification is an attractive alternative to traditional PCR or RT-PCR since thermalcycling is not required, allowing for greater versatility in terms of heating or amplification devices. One such amplification method, termed Loop-Mediated Isothermal Amplification (LAMP) [10], has been optimized for the detection of DNA and/or RNA (RT-LAMP) from a wide range of bacterial and viral pathogens [11], [12], [13], [14], [15], [16], [17], [18], [19], including HIV [20], [21].

LAMP or RT-LAMP exhibits several characteristics that are ideal for integration into a rapid nucleic-acid based diagnostic test. The amplification reaction requires six primers specific for eight separate regions within the target sequence, contributing to the high specificity of the amplification method. Amplified material can typically be detected within 15–60 minutes when incubated at a constant reaction temperature of 60–65°C [22]. LAMP has also proven to be less sensitive to biological inhibitors than PCR [23], [24], which enables direct amplification from clinical specimens, thereby eliminating the need for an additional nucleic acid extraction step. Direct amplification from plasma, whole blood, and oral fluid has previously been demonstrated for HIV-1 [20], [21], [25]. Lastly, immediate visual detection of amplified products is facilitated by the large amount of DNA that is generated by each reaction. Several groups have incorporated fluorescent detection methods into the LAMP assay for real-time or immediate naked-eye detection [15], [17], [21], [22], [26].

The simplicity and isothermal nature of the LAMP procedure opens the door for the evaluation of low-tech integrated devices or novel heating elements, which are appropriate for low-resource settings, where costly equipment and electricity cannot be obtained. In this study, the HIV-1 RT-LAMP assay was evaluated using portable, non-instrumented nucleic acid amplification (NINA) devices that generate heat from the exothermic reaction of calcium oxide and water [27], [28]. We demonstrated the temperature stability of the NINA heating devices and feasibility for POC testing of whole blood specimens from HIV-1 infected individuals.

## Materials and Methods

### Non-instrumented nucleic acid (NINA) heaters

**Figure 1:**
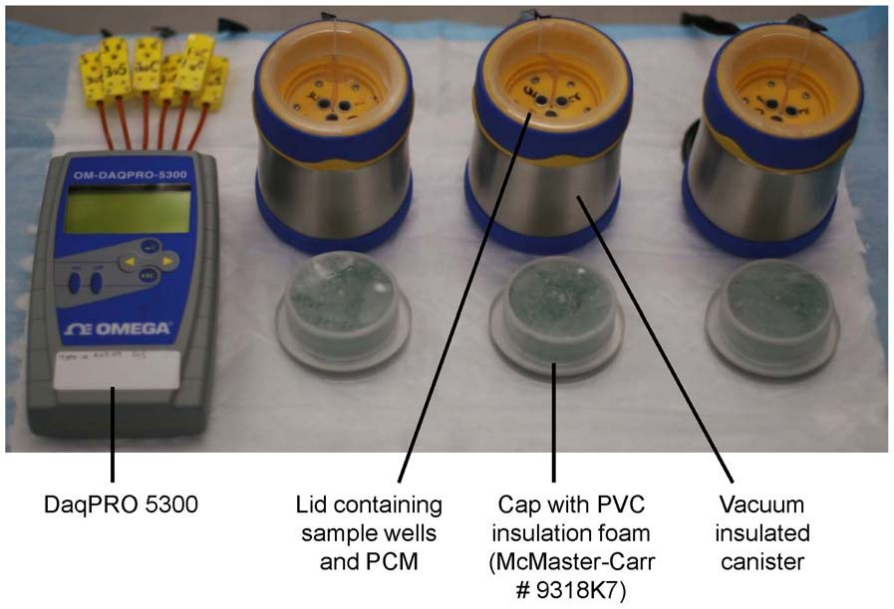
NINA heating devices with DaqPRO 5300 Data recorder.

Prototype NINA heaters were designed and provided by Program for Appropriate Technology in Health (PATH, Seattle, WA), as described [27], [28]. Briefly, an amplification temperature of approximately 60°C was provided by the exothermic reaction of calcium oxide (CaO; Sigma-Aldrich, St. Louis, MO) and water. The heating devices, containing the chemical reaction, were designed using thermally insulated, stainless-steel canisters with plastic screw-top lids (Fig. 1). The lids were modified to contain three sample wells that fit standard 200 µl PCR tubes and were filled with a proprietary phase-change material (PCM) that was used to buffer the heat derived from the exothermic reaction, thereby providing a constant temperature. Lastly, plastic caps containing foam insulation were designed to fit on the top of the canister lids. The thermal profiles of the sample wells were measured and recorded using a digital thermometer (DaqPRO 5300 Data recorder; OMEGA Engineering, Inc., Stamford, CT).

### Linearity panels and clinical specimens

DNA and RNA linearity panels were prepared to determine the sensitivity of the HIV-specific RT-LAMP assay. A DNA panel was generated from DNA extracted from the human monocytic cell line OM-10.1 [29], using a QIAamp DNA blood mini kit (QIAGEN, Valencia, CA). Cell count was used to quantify the input DNA copy number, as a single integrated provirus is contained in each cell [29]. The extracted DNA was diluted ten-fold in RNase-free water to create a linearity panel, ranging from 10^5^ copies/ml to 10^3^ copies/ml. An RNA linearity panel was obtained commercially (PRD801; SeraCare Life Sciences, Milford, MA) and ranged from 2.9×10^6^ copies/ml to 8 copies/ml, as determined by Roche AMPLICOR HIV MONITOR™ v 1.5, Bayer VERSANT HIV-1 RNA bDNA 3.0 Assay, bioMerieux NucliSens® HIV-1 QT, and Abbott Real Time HIV-1 m2000™. RNA was extracted from the panel members using a Viral RNA mini kit (QIAGEN). Negative controls included DNA extracted from PBMC infected with HIV-2 SLRHC [30] and RNA extracted from HIV-2 NIH-Z purified virus (Advanced Biotechnologies Inc., Columbia, MD).

Whole blood from HIV-1 infected individuals was collected as part of a separate, IRB-approved study [31], or obtained commercially (SeraCare Life Sciences). All HIV-positive samples were confirmed using the following tests: Genetic Systems HIV-1/HIV-2 plus O EIA (Bio-Rad Laboratories, Redmond, WA), GS HIV-1 Western blot (Bio-Rad Laboratories), Aptima HIV-1 RNA assay (Gen-Probe, Inc., San Diego, CA), and Amplicor HIV-1 DNA assay (Roche Diagnostics, Branchburg, NJ ). Viral and proviral loads are unknown, since the samples were tested with qualitative, nucleic acid-based assays. All clinical specimens evaluated in this study were obtained from individuals infected with subtype B HIV-1 virus. As a negative control, HIV-1 seronegative blood samples (SeraCare Life Sciences) were included in every experiment involving whole blood. A positive control included HIV-1 seronegative blood spiked with 5×10^6^ virus particles/ml of HIV-1 BaL (Advanced Biotechnologies Inc.).

### RT-LAMP primer design

HIV-1-specific RT-LAMP primers were designed to recognize a conserved sequence within the reverse transcriptase (RT) gene. The six primers required for the RT-LAMP reaction, forward outer (F3), backward outer (B3), forward inner (FIP), backward inner (BIP), and the loop primers (LoopF and LoopB), were designed using the PrimerExplorer V4 software (Eiken Chemical Co. Ltd.; http://primerexplorer.jp/e/). The LAMP primers and amplification cycle have been described in detail by Nagamine et al. [32]. Additional modifications included a linker sequence of four thymidines inserted between the F2 and F1c sequences of the FIP primer, as described [20], and the addition of the fluorescent molecule HEX to the 5′ end of the LoopF primer. The labeled primer, along with a quencher probe, allowed for immediate visual detection of amplified products [21]. The quencher probe consisted of the complementary sequence of the LoopF primer with Black Hole Quencher-1 (BHQ-1) added to the 3′ end. The HIV-1 HXB2 sequence (GenBank accession number AF033819) was used as the reference for generating the RT-LAMP primers. The sequences of the HIV-1 RT-specific primers and quencher are listed in Table 1.

**Table 1:** Sequence of HIV-1 reverse transcriptase-specific RT-LAMP primers.

Primer name	Sequence (5′to 3′)
F3	AGTTCCCTTAGATAAAGACTT
B3	CCTACATACAAATCATCCATGT
FIP	GTGGAAGCACATTGTACTGATATCTTTTTGGAAGTATACTGCATTTACCAT
BIP	GGAAAGGATCACCAGCAATATTCCTCTGGATTTTGTTTTCTAAAAGGC
Loop F	HEX-GGTGTCTCATTGTTTATACTA
Loop B	GCATGACAAAAATCTTAGA
Quencher	TAGTATAAACAATGAGACACC-BHQ1

### RT-LAMP reaction

The RT-LAMP reaction was performed using the following reaction mix: 0.2 µM (final concentration) of each F3 and B3 primers, 1.6 µM of each FIP and BIP primers, 0.8 µM of each LoopF and HEX-LoopB primers, 0.8 M betaine (Sigma-Aldrich), 10 mM MgSO_4_, 1.4 mM dNTPs, 1× ThermoPol reaction buffer (New England Biolabs, Ipswich, MA), 16 U *Bst* DNA polymerase (New England Biolabs) and 2 U AMV reverse transcriptase (Invitrogen, Carlsbad, CA). The reaction was carried out in a total volume of 25 µl for amplification of extracted nucleic acid, 10 µl of which constituted the sample. For amplification of whole blood specimens, a 100 µl reaction volume was used to facilitate visual detection of amplified products. Whole blood was added directly into the reaction at a total volume of 40 µl, following a 1∶4 dilution with red blood cell lysis buffer (2.5 mM KHCO_3_, 37.5 mM NH_4_Cl, and 0.025 mM EDTA), as previously described [21]. The reaction mixture was incubated at 60°C for 60 minutes, using a GeneAmp® PCR System (Applied Biosystems, Foster City, CA) or the NINA heaters. For reactions amplified in the thermalcylcer, an additional two minute heating step of 80°C was added at the end of the amplification cycle to terminate the reaction.

The reaction tubes were evaluated for the presence of amplification, following addition of the quencher probe at a 2∶1 ratio of quencher to labeled-primer, as previously described [21]. Amplification was determined visually by observing fluorescence in the reaction tubes, using the UV lamp from a ChemiDoc XRS system (Bio-Rad Laboratories, Hercules, CA). Amplification was confirmed by electrophoresis using a 1.2% agarose gel containing SYBR® Safe gel stain (Invitrogen), which was subsequently visualized using the ChemiDoc XRS system.

### RT-LAMP in NINA heaters

To compare temperature and amplification consistency, three NINA heaters were tested in parallel. The heating reaction was initiated by adding 18 g of CaO to each NINA canister, followed by 6 ml of water. The lid of each canister was then sealed to contain the exothermic reaction. After adding 200 µl of water to each of the sample wells, temperature recording was initiated. Reaction tubes were added to the sample wells once each reaction chamber reached a temperature of 58.5°C. For all samples incubated in the NINA heater, 15 µl of mineral oil was added to the reaction tube during the reaction mix preparation. The samples were incubated in the heaters for a total of 60 minutes. All reactions were carried out in a temperature-controlled laboratory with an ambient temperature of 28°C, unless otherwise stated. Following the amplification reaction, the samples were incubated for two minutes in a heat block set to 80°C. After each amplification cycle, the temperature profile of each device was analyzed by calculating the temperature mean, standard deviation, median, minimum, and maximum from the data provided by the DaqPRO 5300.

The stability of the NINA heaters at extreme low and high temperatures was evaluated by placing the canisters in a refrigerator set to 4°C or a 37°C incubator during the length of the amplification reaction. The temperature profiles were recorded and compared to those of reactions that occurred at the laboratory room temperature of 28°C.

## Results

### Evaluation of NINA heaters with HIV-specific RT-LAMP assay

To determine the sensitivity of RT-LAMP reaction using RT-specific primers, DNA and RNA linearity panels were tested in a thermalcycler. The limit of detection for HIV-1 DNA was 10 copies/reaction. For the RNA linearity panel, the sample containing 1700 copies/reaction was detected in all of the three replicates, while the sample containing 140 copies/reaction was detected in three out of five replicates (60%). For both DNA and RNA linearity panels, the two samples nearest the limit of detection were chosen to further evaluate the performance consistency between the thermalcycler and NINA heaters. In terms of positivity, the amplification results were consistent between all three heaters and the thermalcycler (Table 2).

**Table 2:** Effect of ambient temperature on amplification efficiency and reproducibility in NINA heaters.

		NINA Heaters								
		28°C			4°C			37°C		
Sample (copies/tube)*	Thermalcycler†	1	2	3	1	2	3	1	2	3
DNA (100)	3/3	+	+	+	+	+	+	+	+	+
DNA (10)	3/3	+	+	+	+	+	+	+	+	+
RNA (1700)	5/5	+	+	+	+	+	+	+	+	+
RNA (140)	3/5	−	+	+	+	+	+	+	+	+

*Calculated by accounting for the volume of each linearity panel member added to the reaction tube.

† Number of positive samples/total number tested.

**Figure 2:**
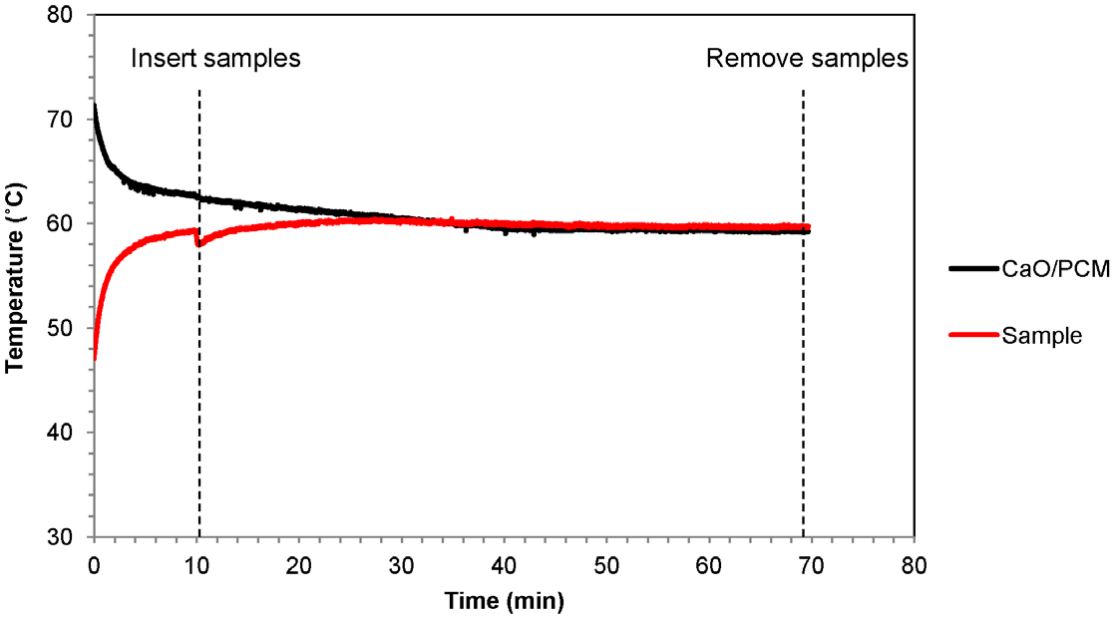
Representative temperature profile of RT-LAMP reaction in NINA heater. The temperature of the reaction tube and phase change material (PCM) was recorded for a 60 minute RT-LAMP reaction using the DaqPRO 5300. Temperature recording was initiated following addition of water to the CaO and terminated upon sample removal.

Since the RT-LAMP assay requires a constant temperature of 60°C for the length of the amplification reaction, the temperature profiles of the sample wells were compared over the course of the incubation and between all three NINA heaters. A representative temperature profile is displayed in Figure 2, showing a steady reaction temperature at or close to 60°C for length of amplification reaction. During the 60 minute incubation, the average temperature for each device was 60.2, 59.8, and 59.7 (Table 3). The minimum temperature achieved during the reaction reflects the fact that the temperature of the sample port dropped temporarily after the sample tubes are added to the device, as shown in Figure 2. The maximum temperature of the devices deviated from the desired reaction temperature of 60°C by less than one degree.

**Table 3:** Effect of ambient temperature on stability and reproducibility of reaction temperature in NINA heaters.

	28°C*			4°C†			37°C†		
	1	2	3	1	2	3	1	2	3
**Mean**	60.2	59.7	59.7	58.3	58.1	58.0	59.8	59.6	59.3
**SD**	0.4	0.5	0.4	1.0	0.7	1.0	0.7	0.5	0.4
**Median**	60.3	59.7	59.7	58.8	58.4	58.4	60.0	59.7	59.5
**Minimum**	58.4	56.9	57.1	55.1	55.9	54.9	57.7	57.8	57.8
**Maximum**	60.9	60.5	60.3	59.4	58.9	59.1	60.6	60.2	59.9

*Values are a mean of 8 independent reactions.

† Values are a mean of 2 independent reactions.

The ability of the NINA heaters to maintain a steady reaction temperature in a wide range of ambient temperatures is essential for POC testing, whether referring to an air-conditioned laboratory or high-temperature field site. To evaluate the performance of the NINA heaters at extreme low or high temperatures, the canisters were placed in a 4°C refrigerator or a 37°C incubator for the length of the amplification reaction. The limit of detection for the DNA and RNA linearity panels was similar to the results obtained in our temperature-controlled laboratory (28°C; Table 2). The greatest degree of temperature variation of the sample wells was observed at the ambient temperature of 4°C (Table 3). The average temperature was approximately two degrees lower than the desired reaction temperature of 60°C. Additionally, the temperature of the devices tended to decline from their steady state during the last 20 minutes of the reaction (data not shown). The temperature profiles at the ambient temperature of 37°C, however, were similar to those at 28°C.

### Validation of NINA heaters with clinical specimens

**Figure 3:**
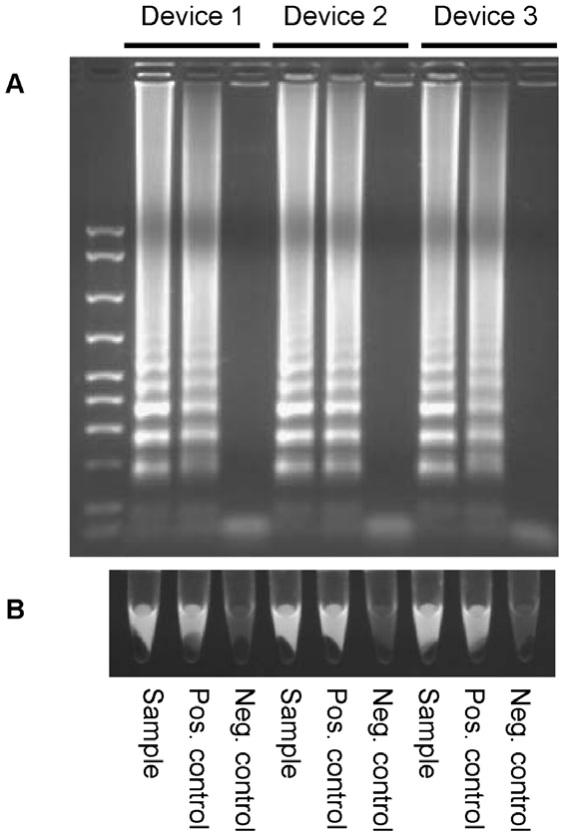
Validation of NINA heaters with clinical specimens. The amplification results from a representative clinical specimen are displayed in the figure. Whole blood from an HIV-1 infected patient was added directly into the RT-LAMP reaction and amplification in all 3 NINA heaters was compared. Amplified material was analyzed by (A) agarose gel electrophoresis or (B) by observing fluorescence in the reaction tubes under a UV light.

Whole blood samples from HIV-1 infected individuals were added directly into the RT-LAMP reaction and tested in the NINA heaters. Positivity of the clinical specimens was consistent between the thermalcycler and devices (Table 4). Amplification consistency was most evident with two of the patient samples (patient #4 and #5) that were only positive in one of the three replicates, regardless of the heating device that was used. All HIV-negative blood samples, included in each reaction, were negative (data not shown). A representative experiment using the NINA heaters is displayed in Figure 3, showing detection by agarose gel and visual identification of fluorescence in the reaction tubes.

**Table 4:** Amplification of HIV-1 DNA/RNA from whole blood in NINA heaters.

	Thermalcycler*	Device 1	Device 2	Device 3
**Patient #1**	3/3	+	+	+
**Patient #2**	3/3	+	+	+
**Patient #3**	3/3	+	+	+
**Patient #4**	1/3	−	−	+
**Patient #5**	1/3	−	+	−
**Patient #6**	3/3	+	+	+

*Number of positive samples/total number tested.

## Discussion

In this study, we demonstrate the performance of portable, inexpensive, non-instrumented nucleic acid (NINA) heaters for amplification of HIV-1 using RT-LAMP. The isothermal amplification reaction coupled with a device that generates heat from an exothermic chemical reaction, as opposed to grid electricity or battery power, comprises a point-of-care NAAT that is practical for use in resource-limited settings. The heating devices require minimal training and technical expertise to operate and take approximately 10–15 minutes to reach a reaction temperature of 60°C once the chemical reaction has been initiated [27], [28]. Furthermore, the temperature of the sample wells remain relatively stable at the desired reaction temperature of 60°C throughout the amplification reaction, as demonstrated by the heating profiles and the consistency in amplification between the devices and thermalcycler.

Since point-of-care testing may refer to an air-conditioned laboratory or a field site with high temperatures and humidity, the stability of the temperature generated by the heating devices must be reliable. Though the temperature profiles at a representative cold temperature of 4°C indicated a loss in reaction temperature towards the end of the 60 minute incubation, the temperature fluctuations were not significant enough to affect the amplification reaction. Regardless, this thermal effect could be mitigated with small modifications to the device to reduce heat loss at lower temperatures. It should be possible to extend the temperature range of the NINA heaters to 4°C and below by either adding a larger quantity of heating mixture, better insulation, or both. Of greater concern is the performance of the NINA heaters in high-temperature field sites, where temperature control is not an option. We demonstrate no difference in the temperature stability of the NINA heaters and amplification consistency at an ambient temperature of 37°C as compared to our temperature-controlled laboratory.

For increased applicability for use at the POC, several modifications can be made to the NINA heaters. The prototype devices evaluated in this study contained only three sample wells; however, up to 16 sample wells can be added to the lid of the insulated canisters for a larger testing volume. In this study, samples were removed from the NINA heaters after the amplification reaction and heated for an additional two minutes in an 80°C heat block to terminate the reaction. While the additional heating step is not necessary to observe the amplified products from extracted nucleic acid, the short, high-temperature incubation facilitates the visual observation of the fluorescent label in the whole blood samples. Modifications may be made to the whole blood sample preparation method to eliminate the need for the heating step. Alternatively, a second temperature-moderating compartment can be added to the alternate end of the NINA canisters, so the samples can be removed from the amplification compartment and reinserted into the 80°C compartment. Lastly, the DaqPRO data recorder was used in this study for validation purposes only and would not be necessary for the final POC product.

The feasibility of using LAMP as a diagnostic method in resource-limited settings has been demonstrated for tuberculosis [33]. To reduce hands-on time and preparation error, the authors describe the use of reaction tubes pre-prepared with lyophilized reaction mix. For POC use, limited sample manipulation and reagent preparation is desired and, therefore, it is anticipated that the test procedure of the end product will include reconstituting the amplification reagents in water and adding the sample directly into the reaction tube. We demonstrate the use of the NINA heaters for amplification directly from whole blood specimens, eliminating the need for a time-consuming, nucleic acid extraction procedure and reducing the volume of sample needed for the amplification reaction. A total volume of 10 µl of whole blood was added to each reaction tube, which can easily be obtained by finger-stick in settings where venipuncture is not feasible. Additionally, our fluorescent detection method enables immediate visualization of amplified products in the absence of specialized equipment. To avoid cross-contamination of amplified material, it is preferred that the reaction tubes remain closed post-amplification. Future modifications will include optimizing the labeled-primer/quencher sequences so that all components can be added into the reaction mix prior to amplification. Due to availability, the Bio-Rad ChemiDoc system was used as the UV source in this study; however, an inexpensive keychain light would be more suitable for naked-eye detection at the POC. For sensitive and specific detection of diverse HIV-1 isolates, including non-B subtypes, identification of the optimal primer set/sets is a key step in the development of the RT-LAMP assay. Although all experiments performed in this study involved subtype B standards and specimens, ongoing research involves the continued development and optimization of RT-LAMP primers based on regions of the HIV-1 genome that are conserved among diverse subtypes. Future studies will include large-scale evaluation of clinical specimens with the optimized RT-LAMP assay and NINA device.

In summary, the RT-LAMP isothermal amplification method used in conjunction with a simplified, chemical heating device exhibits characteristics that are ideal for a rapid NAAT for POC testing. The simplified, portable assay has the potential to fill an important gap in HIV-1 diagnostics, providing immediate knowledge or confirmation of HIV-1 infection status at the POC.
